# The extracellular microscape governs mesenchymal stem cell fate

**DOI:** 10.1186/s13036-016-0037-0

**Published:** 2016-11-21

**Authors:** William J. Hadden, Yu Suk Choi

**Affiliations:** 1 University of Sydney Medical School & Kolling Institute of Medical Research, Sydney, NSW Australia; 2 School of Anatomy, Physiology and Human Biology, University of Western Australia, Entrance 2, Hackett Dr, M309, Level 1, Crawley, WA 6009 Australia

**Keywords:** Extracellular matrix, Mechanotransduction, Adhesion, Stem cell differentiation

## Abstract

Each cell forever interacts with its extracellular matrix (ECM); a stem cell relies on this interaction to guide differentiation. The stiffness, nanotopography, protein composition, stress and strain inherent to any given ECM influences stem cell lineage commitment. This interaction is dynamic, multidimensional and reciprocally evolving through time, and from this concerted exchange the macroscopic tissues that comprise living organisms are formed. Mesenchymal stem cells can give rise to bone, cartilage, tendon and muscle; thus attempts to manipulate their differentiation must heed the physical properties of incredibly complex native microenvironments to realize regenerative goals.

## Background

Stem cell fate was long assumed to rely solely on biochemical messengers, yet the story of cellular differentiation is now proving much more intricate than initially theorized. As early as 1898 Councilman described cellular chemotaxis in the setting of acute interstitial nephritis and postulated that soluble substances in certain locations were responsible for the development of lymphoid foci [1]. Later studies in the 1970’s identified biochemical factors responsible for cellular migration in cancer models [2], and further research in the following decade described the molecular signals necessary for the induction of differentiation in T cells and glial progenitors, respectively [3, 4]. Still later studies demonstrated the powerful capacity of a biologically-inspired chemical signal gradient to induce cellular migration and differentiation [5–7]. Because of this pattern of accumulated knowledge, the potential for biochemical signals to induce stem cell migration, differentiation and proliferation has been appreciated for many years and early intimations regarding mechanically-directed behaviour drew less notice [8].

Although historically less recognized, clues to the importance of physical properties in tissue development are ubiquitous in nature. Life uses malleable muscles to lever tendons across rigid joints, it creates discreet neuromuscular junctions from tissues with markedly different physical properties and it protects an intricate, delicate neural apparatus with rigid bony structure. But perhaps most relevant to regenerative medicine, pathophysiological states are often defined by an altered mechanical microenvironment. Gliosis following a cerebrovascular insult, fibrosis after a myocardial infarction and collagen deposition after epithelial disruption all forever alter the mechanical microenvironment experienced by individual cells resident to those tissues [9]. This shifting mechanical microscape makes it all the more necessary for therapies intent on rectifying pathology to prepare for the inductive properties inherent to the pathophysiological environments they will encounter.

Specifically, the elasticity [10–13], nanotopography [14, 15], protein composition [16, 17], and mechanical strain [18] inherent to the extracellular matrix (ECM) are all independent factors guiding stem cell lineage commitment. Even more fascinating is recent work suggesting stem cells retain mechanical memory for surfaces previously encountered [19]. This has striking implications not only for the inherent validity of the conclusions drawn from studies using in vitro models, but on the understanding of the in vivo temporal changes potentially implicit in aging. Understanding and deconstructing this complex menu of signals is core among the current challenges faced by regenerative medicine as each ECM inflicts a specific array of stressors on its resident cells. Factors such as cell density, shape and size [20–22], as well as the degree and type of cell-cell interaction and the dimensionality experienced within each tissue further complicate attempts to recapitulate these environments ex vivo*.*

The implications for understanding the interaction between cells and their respective ECMs are critical and far-reaching. The hopeful search for panacea in many medical fields has focused significant energy on stem cell therapy; from basic science to randomized clinical trials [23–25]. While seemingly safe in their application [26], medicine is marginally closer to sound and applicable stem cell therapies in regenerative medicine [27, 28]. There are of course many reasons this large cumulative effort has progressed slowly toward clinical resolution. In part, bureaucratic engines always run slower than scientific minds and funding for clinical trials in this field is doubly wrought with financial and political mines [29]. The emergent and rapidly expanding use of adult stem cell sources has softened some of the political stigma attached to the prefix ‘embryo’. Still, our lack of understanding and our inability to control all biochemical and biomechanical inputs that direct stem cell fate is certainly rate-limiting. The global sum of all signals encountered by a differentiating cell, whether chemical or physical, past or present, act as one unique set of circumstances directing the commitment of each cell toward a specific lineage [30]. Nature has replicated these exquisite circumstances with innumerable diversity for millennia; proof that such discretion is possible.

Mesenchymal stem cells (MSCs) are easily accessible progenitors that can give rise to fat, muscle, tendon, cartilage and bone [31, 32]. The promise of a tool to combat some of the most burdensome human pathologies, including myocardial infarction, osteoporosis and osteoarthritis, makes this stem cell source particularly appealing to biomedical engineers [33]. The future of medicine for an aging population may well depend on our ability to understand and control those slow and seemingly inevitable processes that alter physical structures. This review explores the guiding role of the ECM in MSC differentiation and discusses attempts to deconstruct and translate these ECM component signals for tissue engineering in the search for regenerative therapies.

## The physical world

### Stiffness

**Fig. 1:**
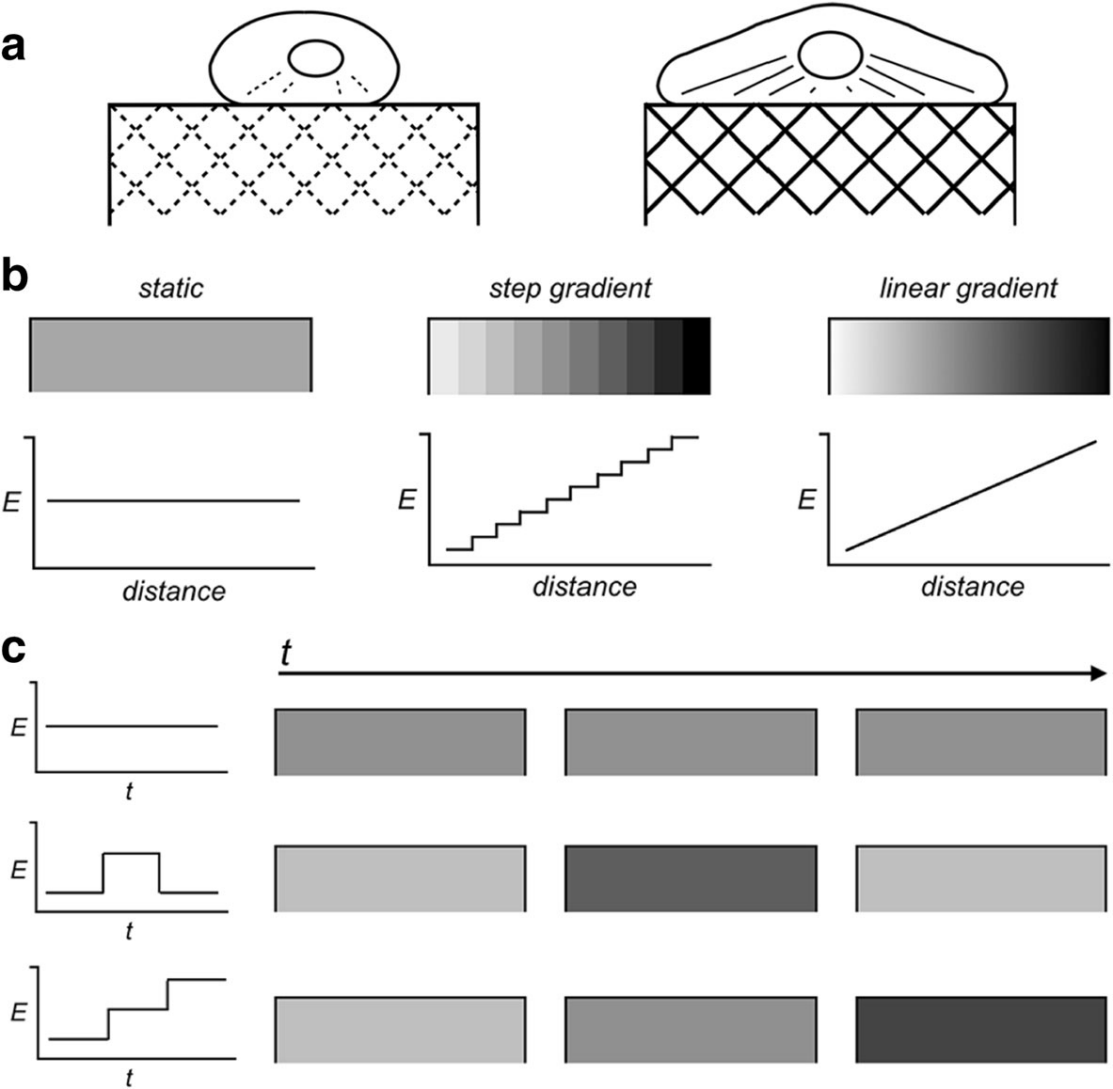
Schematic representation of static and dynamic ECM stiffness. **a** stiffness influencing stem cell behaviour (soft on the left and stiff on the right); (**b**) spatial dynamic; and (**c**) time dynamic changes of ECM stiffness (*E*: Young’s Modulus)

The stiffness of infarcted fibrous heart tissue is much greater than that of the adjacent healthy myocardium [9, 34]. This clear physical demarcation has so far complicated the ability of introduced stem cell therapies to remodel and replace infarcted myocardium. Indeed ECM stiffness (Young’s modulus) has been shown to influence spreading [35, 36], migration [10], proliferation [37] and differentiation [17, 38] in multiple stem cell lineages [11, 12], and is a key physical property guiding stem cell behaviour (Fig. 1a). Tissue stiffness not only dictates differentiation, but informs cytoskeletal arrangement on a macro scale. Myotubes form myosin/actin striations preferentially on those substrates that mimic the passive stiffness of normal muscle [13, 39]. This is also true of artificial environments that mimic rigid collagenous bone and soft neural tissue; where the absolute stiffness in isolation directs osteogenic and neurogenic differentiation respectively [11]. This realization has led to the pursuit of substrates whose stiffness can be modulated in both two [40] and three [41] dimensions under static or dynamic conditions.

Collateral to dimensionality, efforts to create stiffness gradients in both space (Fig. 1b) and time (Fig. 1c) have added further pieces to our most accurate models of in vivo stiffness conditions. Controlling stiffness gradients in spatial planes has been accomplished with a variety of methods, including repeated freeze-thaw cycles with liquid nitrogen and cylindrical polyvinyl alcohol columns [42], heat gradients within polymerizing polydimethylsiloxane [37], polyelectrolyte monolayers with patterned cross-linker [43], inclusion of rigid particles in a soft hydrogel [44], and microfluidic mixing of different polyacrylamide (PA) solutions. Still other methods have employed photo-initiators and a patterned [40, 45, 46] or moving [47–49] photomask overlying a polymerization chamber to create stiffness gradients. However, tissues are not static in time. Temporal control over synthetic substrate stiffness is required to fully appreciate and simulate in vivo environments [50]. Early successful attempts at dynamic control over substrate stiffness came using photo-responsive elements embedded within those substrates [51–53], or chemicals whose disparate molecular weights stiffened hyaluronic acid hydrogels [54]. This approach allowed external control over substrate stiffness and gave researchers license to guide chondrogenic differentiation with specific timing or mimic the development of mature cardiomyocytes from mesodermal precursors. Building from this approach, near-infrared photons used to excite embedded gold nanorods have allowed researchers to write custom stiffness patterns into synthetic hydrogels [55], and still others have developed hydrogels that respond to their biological environment by releasing metalloproteinase (MMP) inhibitors in response to the activity of that enzyme group [56].

Just as tissues are not static in time, nor are they two-dimensional. As methods to build three-dimensional models of the ECM become more sophisticated, gains that moved this field from static to dynamic to bio-responsive are being incorporated into plant-inspired, 3D-printed hydrogels that not only respond to the chemical signals of in vivo environments (i.e. MMPs) but to the very physical properties resident cells are feeling [57].

### Stress & strain

**Fig. 2:**
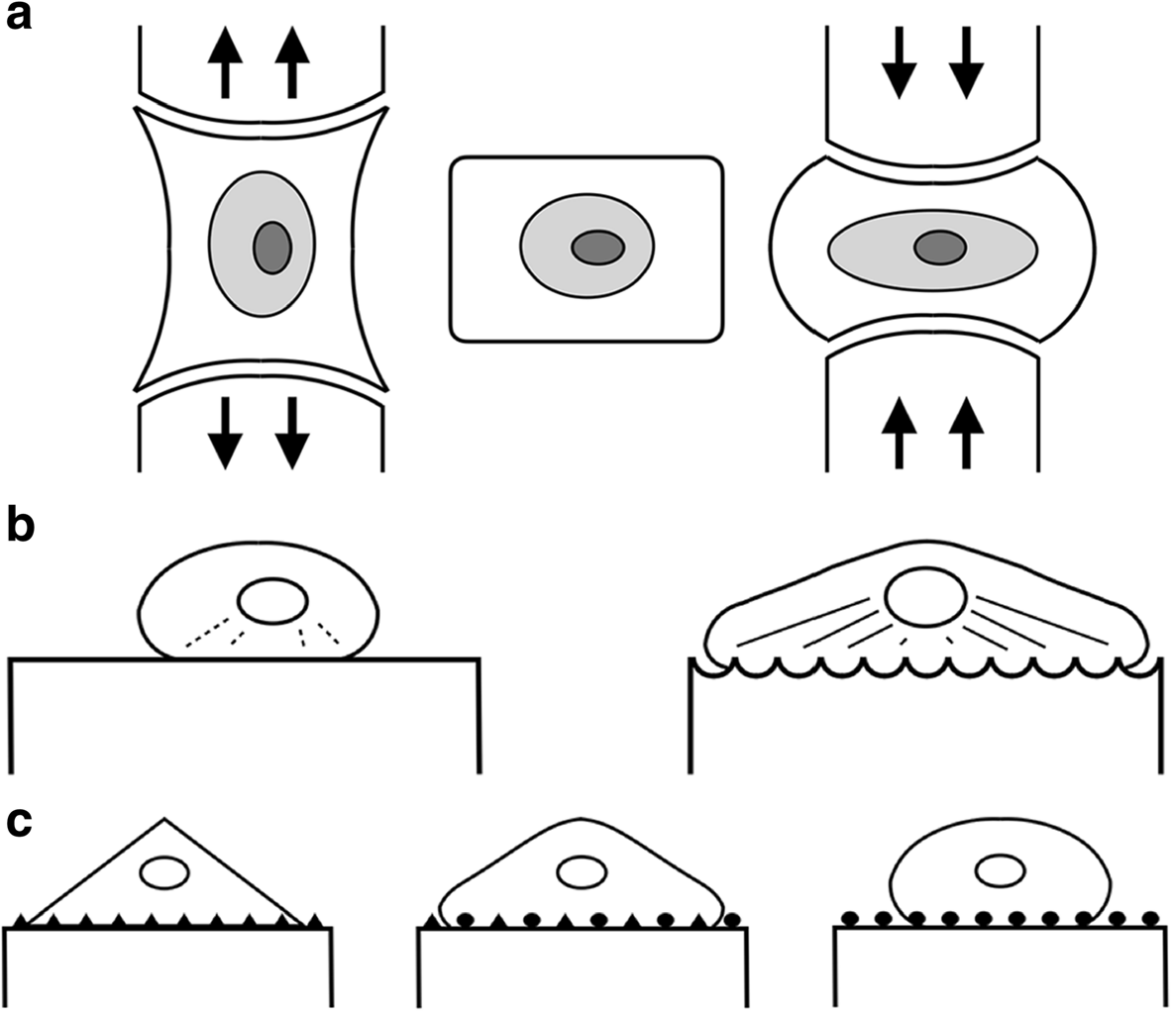
Schematic representation of (**a**) stress & strain; (**b**) nano- or micro-topography; and (**c**) protein composition or arrangement influencing stem cell interaction with the ECM

Lungs expand due to relative compartmental pressure changes and recoil due to the innate elasticity of their interstitium; hearts beat in rhythmic contraction-relaxation cycles; tendons and cartilage bear the longitudinal and compressive forces generated by ambulation. Thus the strain and stress applied to culture environments are important considerations for ECM models (Fig. 2a). Early studies with MSCs showed that uniaxial strain will induce transient increases in collagen I expression and alignment along that axis [58]. Further, a 3-5% tensile strain on a collagen I substrate can induce osteogenesis in MSCs [59], and biaxial stretching systems can influence alignment of MSCs via mechanosensing [60]. Following from this, the imposition of cyclical tensile strain to mimic the loading and unloading of the skeletal system was shown to promote osteogenesis and angiogenesis on three-dimensional collagen I scaffolds [61]. Conversely, applying a coordinated tensile strain coupled with electrical stimulation on a decellularized porcine myocardial ECM scaffold sped up myocardial differentiation of MSCs seven-fold [62]. MSCs co-cultured with intervertebral disc cells will readily undergo chondrogenic differentiation under cyclical compressive force [63]. Similarly, cyclic hydrostatic pressure (mimicking compression) has also induced chondrogenesis in MSCs [64], and compression in the form of applied gravity and low-density ultrasound have restored collagen content and mineralized bone via osteogenic differentiation of MSCs [65, 66].

These fascinating observations are testament to the shear economy of differentiation; all available inputs are synthesized to trigger fate decisions. Even fluid shear stress modulated by β1 integrin and experienced by many tissues in the body, has shown particular applicability to MSC osteogenic differentiation [67]. Further, these effects are not static or one-directional, as the ability of MSCs to deform the ECM around them differs depending on developmental stage of those MSCs. Fetal, neonatal and adult heart tissue all respond differently to ECMs that are evolving in composition through time [68]. This implies that although inanimate, there is a developmental dependency between stem cells and their ECM; a reciprocal guidance developing in concert.

### Nanotopography

The spicules are thinner and the spaces between them greater in osteoporotic trabecular bone. This altered environment imposes new distance, surface area and volume parameters on its residents (Fig. 2b), and we now know that this altered nanotopography also directs MSC fate (aligning electrospun nanofibers in a synthetic matrix will force increased mineralization of differentiating osteoblasts) [69]. Concordantly, nanofibers mimicking the pattern of cartilage ECM encourages chondrogenesis [70]. The fascinating detail with which stem cells use their nanoenvironment to acquire cues is perhaps best exemplified in by results showing that the mere addition of Si-OH groups to a matrix scaffold can induce osteogenesis of MSCs in the absence of growth signal supplementation [71]. Thus, not all cues are either biochemical or mechanical; some exist at the boundary between and the causality of their inductive property is less clear. The gross physical pattern of the ECM can also provide a constraining environment thus allowing more cytoskeleton tension within MSCs, which in turn leads to greater osteogenic differentiation [72], and the addition of a flow stimulus (albeit crossing into the realm of shear forces) through specific nanotopography modulates fibrochondrogenesis of MSCs [73]. Lastly, Yang et al. best exemplified the intertwined nature of three-dimensional microenvironment and the forces that environment imposes on resident cells with their assays of stem cell mechanobiology on substrates with built-in elastomeric microposts [15]. This last citation relays the inherent difficulty in deconstructing component physical cues of a given microenvironment.

### Protein composition

Skeletal muscle relies on an ECM dominated by laminin while bone tissue is replete with collagen-containing ECM. This stark difference between two descendants of mesoderm tells us that ligand composition, density and spatial arrangement is consequential (Fig. 2c). Undoubtedly, the interaction between stem cells and the ECM is bidirectional and geography-dependent, so the potential for interaction (i.e. the availability of binding sites) impacts heavily on the ultimate fate of stem cells exposed to these environments [16]. Specifically, the integrin density connecting cells to the ECM will fate different lineage commitments for MSCs residing in adipose or skeletal tissue and helps in part to explain why adipogenesis proceeds despite the induction of osteogenic stress in adipose tissue [74]. The density and spacing of the cell-adhesive tripeptide arginine-glycine-aspartate (RGD) can influence MSC fate, and for some time the tethering of collagen linkage points across inherent substrate pores was thought to guide differentiation (although this latter point is becoming somewhat contentious) [62, 75, 76]. Finally, the nature of the ligand itself can factor in the outcome of MSC differentiation; hyaluronic acid (HA) has been shown to induce chondrogenesis and chondroitin sulphate (CS) has been shown to induce osteogenesis [77]. Analogous to previously discussed silicon-hydroxide group addition and perhaps most intriguing of all, is the ability of matrices to offer physical binding sites for chemical and hormonal signals (in this case melatonin), thus acting as reservoirs that blur boundaries between biomechanical and biochemical induction of differentiation [78–80].

## Rebuilding the native microenvironment

**Fig. 3:**
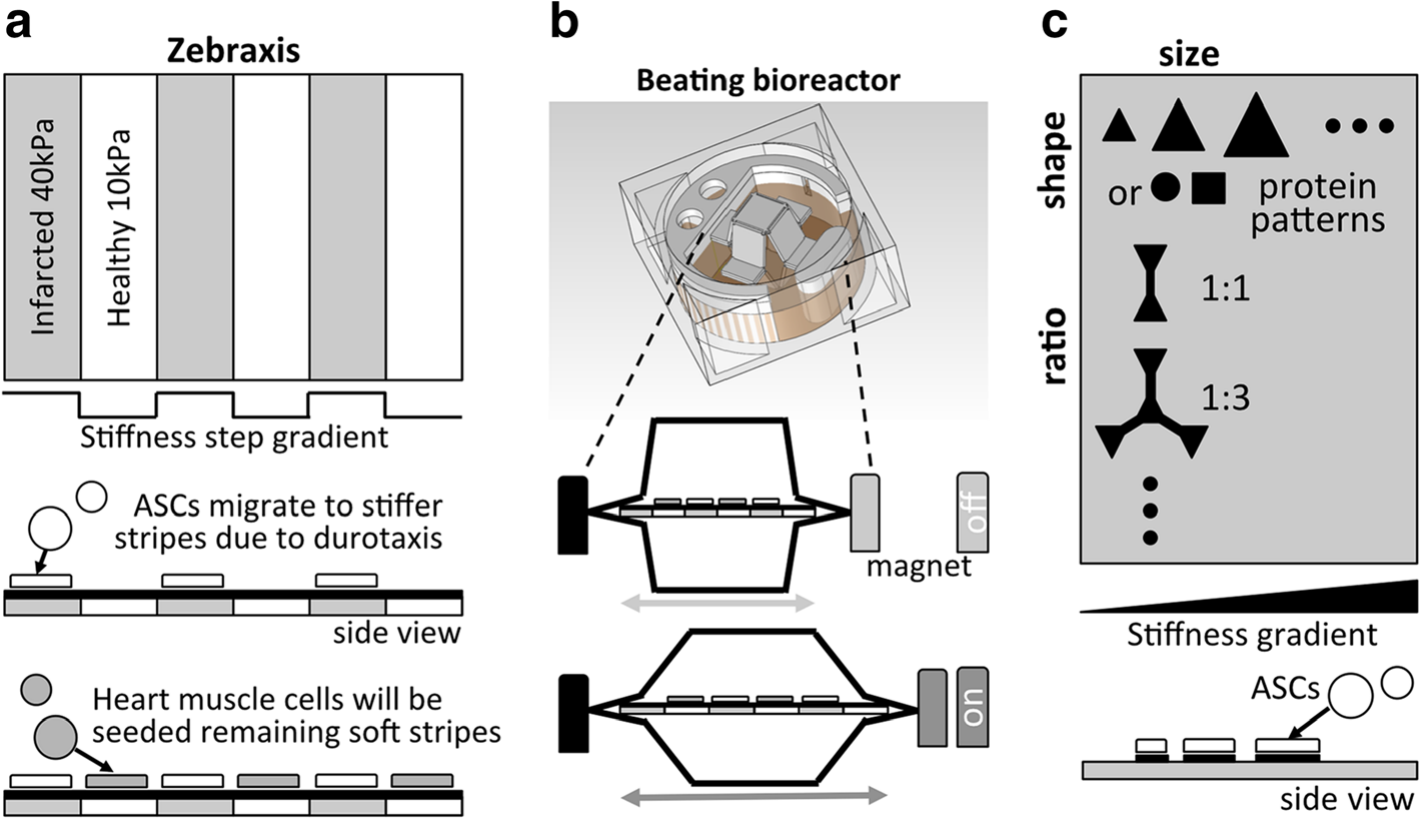
Schematic model of a physical environment encountered by adipose-derived stem cells (ASCs) in infarcted myocardium; (**a**) discrete stiffness changes; (**b**) dynamic strain; and (**c**) gradients in stiffness, shape/size and protein composition

Tissue engineering is now faced with the challenge of how to most accurately and reliably reproduce and modulate each individual physical factor and then combine them into one cohesive environment. The enormity of this task is immediately realized when, for example, we attempt to faithfully reconstruct the physical changes wrought by myocardial infarction and seed those environments with adipose-derived stem cells. Discrete stiffness changes (Fig. 3a) coupled to an actively beating bioreactor (Fig. 3b) must be platformed on multiple gradients of stiffness, nanotopography and protein composition (Fig. 3c) in both two and three dimensions. The shear complexity of this deconstructive approach therefore also demands the exploration of endogenous ECMs to satisfy this same purpose. Indeed, recent studies have shown that the matrix laid down by bone marrow- or adipose-derived stem cells promotes the growth of those cell lines more so than other cell types, even after decellularization and reseeding. To this end, the induction of small, high frequency deformations in osteogenic cultures can elicit more efficient production of ECM for MSC differentiation [81]. Further, these biologically synthesized matrices promote differentiation to the lineages that originally laid them down [82]. Even artificial three-dimensionally-printed scaffolds seeded with differentiated cells of a specific tissue (which are subsequently removed) for the sole purpose of decorating that artificial matrix with biological ECM, create more effective inductive matrices for MSCs [83, 84]. An artificial collagen-polyglyconate scaffold seeded with smooth muscle and urothelial cells has generated bladder tissue sufficient for transplantation [27] and decellularized trachea has been recolonized by recipient epithelial cells after implantation [28]. Additional recommended reviews in this field further explore the use of decellularized or naturally-derived ECMs [85–88]. While the collaborative regenerative power of resident cells in native environments is clear, this research affirms the idea that 3D printing with intricately designed biocompatible materials may offer promising solutions as these technologies converge upon common applications [57].

## Regenerative goals

### Muscle

In the wake of much promise, early studies in cardiomyocyte differentiation showed MSC osteoblastic differentiation when exposed to infarcted fibrous heart tissue [9]. Although clinically disappointing, this outcome was scientifically interesting and correlated with findings regarding the absolute stiffness differences inherent to infarcted heart tissue and healthy myocardium [34]. This work served to punctuate the importance of local tissue stiffness in cardiac stem cell therapy and heralded the need for a deconstructive approach to better understand the consequences of the individual forces experienced by stem cells in vivo*.* Consequent of this realization, researchers have recreated functional myotubes on striped surfaces of alternating stiffness [12, 89], and shown that myoblast differentiation is delayed in the setting of fibrosis [90]. The capability to reintegrate this knowledge with therapy is now beginning to take form. Jeffords and colleagues have developed cardiac matrix hydrogels using a genipin cross-linker to more accurately mimic the stiffness conditions favourable for endothelial revascularization of infarcted heart tissue [54, 91, 92]. Interestingly, the application of an acellular ECM substitute composed of high stiffness hyaluronic acid provided better cardiac function after myocardial infarction (MI). In this case, simply altering a pathological ECM allowed resident cells to effect superior outcomes [93].

### Tendon/ligament

MSC enhancement of potential tendon and ligament grafts has centred on cyclical tension of a collagen scaffold either artificially produced or decellularized from a donor site, then re-impregnated with MSCs to promote fibroblastic differentiation [94, 95]. This type of loading elicited uniaxial alignment of MSCs on synthetic yarns and promoted the production of collagen I [96], and a 3 % strain induced tendon-like gene expression profiles in MSCs seeded on these scaffolds [97]. Tendon regeneration also exemplifies the extraordinary impact of as yet unknown resident biomolecular cues [98]. The leap from decellularized donor tissue to wholly synthetic grafts hinges on this understanding [99], and highlights the limitations of synthesizing a tissue with incomplete knowledge of its components. Current steps in this direction employ co-electrospinning to form synthetic anterior cruciate ligament ECM with in-built mechanical gradients for better integration when implanted [100], or the application of combined mechanical loading of a 3D ECM model to regenerate periodontal ligaments [101].

###  Cartilage

Existing at relatively unpredictable biological junctions, cartilaginous tissue is a model for the combined impact of tensile, shear, compressive and stiffness conditions experienced by biological tissues and transmitted through their respective ECMs. These component parts have been deconstructed with some success and slowly reintegrated to form a broader understanding of this tissue’s development. Mimicking the native environment experienced by cartilaginous tissue with cyclic hydrostatic pressure loading increases chondrocyte differentiation of BMSCs [102–105]. Concurrent with these results, cartilaginous differentiation is also upregulated when MSCs are mechanically compressed in a gel matrix [106], or forced to compress under low intensity pulsed ultrasound [107]. As mentioned earlier, distortional stress analogous to artificial gravity in a centrifuge system can increase proliferation and proteoglycan/collagen II production in an MSC population subjected to these conditions [108]. Finally, an amalgamation of tensile, shear and compressive forces meant to replicate joint stress and strain has been shown to guide MSCs toward chondrogenesis [109]. Just as tendon ECM serves as an example of the importance of biophysical and biomolecular integration of signals, cartilage ECM reveals the importance of various interacting mechanical inputs required for proper differentiation.

### Bone

With osteogenic potential, bone regeneration is an obvious important application for MSCs. Stiffness [11], mechanical loading [110] and three-dimensional pore size [111] all impact osteogenic differentiation. When considered, the nature of anatomical progression from cortical to cancellous bone and the repetitive load-bearing function of this tissue would hypothesize as much. To investigate these ends, intermittent shear stress and cyclic hydrostatic pressure have been shown to elicit mechanosensitive gene profiles in MSCs and further work has shown enhanced production of bone ECM using pulsatile versus fixed hydrostatic shear flow [112, 113]. Interestingly, high acoustic frequencies will also guide MSCs toward osteogenesis and spare the adipogenic induction seen at lower frequencies [114], and larger three-dimensional pore size will elicit more osteogenic differentiation [111]. Analogous to earlier lessons unveiled by Brietbach et al., endocardial valve ossification has been observed when MSCs encounter the required inductive mechanical stress [9, 115]. This further highlights the crossing and complicated differentiation pathways that evolve from MSC therapy under subtly different mechanical circumstances and emphasizes the importance of applying negative results to a broader paradigm.

Fascinatingly, MSC osteogenesis illuminates a far-reaching implication of the dynamic interaction between stem cells and their ECM: aged MSCs tend to mineralize their environment at a slower rate under the same mechanical circumstances as their younger counterparts because of a reduced ability to undergo mechanotransduction [116]. This interaction implies a critical feedback loop and cyclical regression of bone density with age. Considering that osteogenesis from an MSC derivative population is greatly enhanced after injury when titanium implants are coated with a collagen matrix [117], current joint replacement or fracture fixation techniques may benefit from bio-regenerative therapy just as they will be limited by the endogenous capacity of aging MSCs to facilitate concordant repair.

## Conclusions

Clearly the physical environment experienced by a resident mesenchymal stem cell through its ECM has much to say regarding the ultimate fate of that cell. Absolute stiffness (as well as patho- or physiological gradients in ECM stiffness) guides development and dictates response to a disease insult. Nanotopography can blur the boundary between geography and the physical forces imposed by that microenvironment, and ligand density can similarly blend biochemical signals with biomechanical ones. Stress and strain experienced cyclically (heart), sporadically (tendon) or constantly (bone) inform the modelling, alignment and economy of materials in those respective tissues. Further, all of these ECM components evolve through time and are reciprocal in their interaction with resident cells: osteoblasts can form matrix that stiffens their environment leading to further osteogenic differentiation; the formation of functional myotubes leads to concerted beating that exacerbates the cyclical stress imposed on that tissue; native alignment of tendon tissue informs future recruits to that tissue as to the appropriate function therein; and the decoration and incorporation of growth signals in developing bone ensures signals for future bone regeneration [98].

Effective regenerative medicine requires exquisite models of the ECM; models that account for the infinitesimal fractional inputs accumulated across billions of years of life interacting with its environment. The ruthless economy of evolution demands that whether biochemical or mechanical, past or present, static or dynamic, no force or dimension or bond is unnecessary. Thus tissue engineering is proving complex beyond imagination, but its stated goals are not unattainable.

## References

[1] Councilman WT (1898). Acute Interstitial Nephritis. J Exp Med.

[2] Hayashi H, Yoshida K, Ozaki T, Ushijima K (1970). Chemotactic factor associated with invasion of cancer cells. Nature.

[3] Bach JF, Papiernik M (1981). Cellular and molecular signals in T cell differentiation. Ciba Found Symp.

[4] Raff MC, Lillien LE (1988). Differentiation of a bipotential glial progenitor cell: what controls the timing and the choice of developmental pathway?. J Cell Sci Suppl.

[5] Li Jeon N, Baskaran H, Dertinger SK, Whitesides GM, Van de Water L, Toner M (2002). Neutrophil chemotaxis in linear and complex gradients of interleukin-8 formed in a microfabricated device. Nat Biotechnol.

[6] Liu X, Shi S, Feng Q, Bachhuka A, He W, Huang Q (2015). Surface Chemical Gradient Affects the Differentiation of Human Adipose-Derived Stem Cells via ERK1/2 Signaling Pathway. ACS Appl Mater Interfaces..

[7] Arnold SJ, Robertson EJ (2009). Making a commitment: cell lineage allocation and axis patterning in the early mouse embryo. Nat Rev Mol Cell Biol.

[8] Steinberg MS (1962). Mechanism of tissue reconstruction by dissociated cells. II. Time-course of events. Science.

[9] Breitbach M, Bostani T, Roell W, Xia Y, Dewald O, Nygren JM (2007). Potential risks of bone marrow cell transplantation into infarcted hearts. Blood.

[10] Lo CM, Wang HB, Dembo M, Wang YL (2000). Cell movement is guided by the rigidity of the substrate. Biophys J.

[11] Engler AJ, Sen S, Sweeney HL, Discher DE (2006). Matrix elasticity directs stem cell lineage specification. Cell.

[12] Choi YS, Vincent LG, Lee AR, Dobke MK, Engler AJ (2012). Mechanical derivation of functional myotubes from adipose-derived stem cells. Biomaterials.

[13] Engler AJ, Griffin MA, Sen S, Bonnemann CG, Sweeney HL, Discher DE (2004). Myotubes differentiate optimally on substrates with tissue-like stiffness: pathological implications for soft or stiff microenvironments. J Cell Biol.

[14] Fu J, Wang YK, Yang MT, Desai RA, Yu X, Liu Z (2010). Mechanical regulation of cell function with geometrically modulated elastomeric substrates. Nat Methods.

[15] Yang MT, Fu J, Wang YK, Desai RA, Chen CS (2011). Assaying stem cell mechanobiology on microfabricated elastomeric substrates with geometrically modulated rigidity. Nat Protoc.

[16] Luo W, Chan EW, Yousaf MN (2010). Tailored electroactive and quantitative ligand density microarrays applied to stem cell differentiation. J Am Chem Soc.

[17] Flanagan LA, Rebaza LM, Derzic S, Schwartz PH, Monuki ES (2006). Regulation of human neural precursor cells by laminin and integrins. J Neurosci Res.

[18] Saha S, Ji L, de Pablo JJ, Palecek SP (2006). Inhibition of human embryonic stem cell differentiation by mechanical strain. J Cell Physiol.

[19] Yang C, Tibbitt MW, Basta L, Anseth KS (2014). Mechanical memory and dosing influence stem cell fate. Nat Mater.

[20] Gao L, McBeath R, Chen CS (2010). Stem cell shape regulates a chondrogenic versus myogenic fate through Rac1 and N-cadherin. Stem Cells.

[21] McBeath R, Pirone DM, Nelson CM, Bhadriraju K, Chen CS (2004). Cell shape, cytoskeletal tension, and RhoA regulate stem cell lineage commitment. Dev Cell.

[22] Kilian KA, Bugarija B, Lahn BT, Mrksich M (2010). Geometric cues for directing the differentiation of mesenchymal stem cells. Proc Natl Acad Sci U S A.

[23] Hare JM, Traverse JH, Henry TD, Dib N, Strumpf RK, Schulman SP (2009). A randomized, double-blind, placebo-controlled, dose-escalation study of intravenous adult human mesenchymal stem cells (prochymal) after acute myocardial infarction. J Am Coll Cardiol.

[24] Lee PH, Kim JW, Bang OY, Ahn YH, Joo IS, Huh K (2008). Autologous mesenchymal stem cell therapy delays the progression of neurological deficits in patients with multiple system atrophy. Clin Pharmacol Ther.

[25] Ning H, Yang F, Jiang M, Hu L, Feng K, Zhang J (2008). The correlation between cotransplantation of mesenchymal stem cells and higher recurrence rate in hematologic malignancy patients: outcome of a pilot clinical study. Leukemia.

[26] Lalu MM, McIntyre L, Pugliese C, Fergusson D, Winston BW, Marshall JC (2012). Safety of cell therapy with mesenchymal stromal cells (SafeCell): a systematic review and meta-analysis of clinical trials. PLoS One.

[27] Atala A, Bauer SB, Soker S, Yoo JJ, Retik AB (2006). Tissue-engineered autologous bladders for patients needing cystoplasty. Lancet.

[28] Macchiarini P, Jungebluth P, Go T, Asnaghi MA, Rees LE, Cogan TA (2008). Clinical transplantation of a tissue-engineered airway. Lancet.

[29] Couture LA, Carpenter MK (2015). 2005 Donor Eligibility Requirements: Unintended Consequences for Stem Cell Development. Stem Cells Transl Med.

[30] Discher DE, Janmey P, Wang YL (2005). Tissue cells feel and respond to the stiffness of their substrate. Science.

[31] Pittenger MF, Mackay AM, Beck SC, Jaiswal RK, Douglas R, Mosca JD (1999). Multilineage potential of adult human mesenchymal stem cells. Science.

[32] Mackay AM, Beck SC, Murphy JM, Barry FP, Chichester CO, Pittenger MF (1998). Chondrogenic differentiation of cultured human mesenchymal stem cells from marrow. Tissue Eng.

[33] Statistics CoA, Australian Bureau o. National Health Survey. document. Commonwealth of Australia; Australian Bureau of Statistics, 2015 2015-12-08. Report No

[34] Berry MF, Engler AJ, Woo YJ, Pirolli TJ, Bish LT, Jayasankar V (2006). Mesenchymal stem cell injection after myocardial infarction improves myocardial compliance. Am J Physiol Heart Circ Physiol.

[35] Engler A, Bacakova L, Newman C, Hategan A, Griffin M, Discher D (2004). Substrate compliance versus ligand density in cell on gel responses. Biophys J.

[36] Pelham RJ, Wang Y (1997). Cell locomotion and focal adhesions are regulated by substrate flexibility. Proc Natl Acad Sci U S A.

[37] Wang PY, Tsai WB, Voelcker NH (2012). Screening of rat mesenchymal stem cell behaviour on polydimethylsiloxane stiffness gradients. Acta Biomater.

[38] Deroanne CF, Lapiere CM, Nusgens BV (2001). In vitro tubulogenesis of endothelial cells by relaxation of the coupling extracellular matrix-cytoskeleton. Cardiovasc Res.

[39] Levy-Mishali M, Zoldan J, Levenberg S (2009). Effect of scaffold stiffness on myoblast differentiation. Tissue Eng Part A.

[40] Tse JR, Engler AJ (2010). Preparation of hydrogel substrates with tunable mechanical properties. Curr Protoc Cell Biol.

[41] Pek YS, Wan AC, Ying JY (2010). The effect of matrix stiffness on mesenchymal stem cell differentiation in a 3D thixotropic gel. Biomaterials.

[42] Kim TH, An DB, Oh SH, Kang MK, Song HH, Lee JH (2015). Creating stiffness gradient polyvinyl alcohol hydrogel using a simple gradual freezing-thawing method to investigate stem cell differentiation behaviors. Biomaterials.

[43] Hopp I, Michelmore A, Smith LE, Robinson DE, Bachhuka A, Mierczynska A (2013). The influence of substrate stiffness gradients on primary human dermal fibroblasts. Biomaterials.

[44] Kuo CH, Xian J, Brenton JD, Franze K, Sivaniah E (2012). Complex stiffness gradient substrates for studying mechanotactic cell migration. Adv Mater.

[45] Nemir S, Hayenga HN, West JL (2010). PEGDA hydrogels with patterned elasticity: Novel tools for the study of cell response to substrate rigidity. Biotechnol Bioeng.

[46] Wong JY, Velasco A, Rajagopalan P, Pham Q (2003). Directed movement of vascular smooth muscle cells on gradient-compliant hydrogels. Langmuir.

[47] Johnson PM, Reynolds TB, Stansbury JW, Bowman CN (2005). High throughput kinetic analysis of photopolymer conversion using composition and exposure time gradients. Polymer.

[48] Marklein RA, Burdick JA (2010). Spatially controlled hydrogel mechanics to modulate stem cell interactions. Soft Matter.

[49] Sunyer R, Jin AJ, Nossal R, Sackett DL (2012). Fabrication of hydrogels with steep stiffness gradients for studying cell mechanical response. PLoS One.

[50] Burdick JA, Murphy WL (2012). Moving from static to dynamic complexity in hydrogel design. Nat Commun.

[51] DeForest CA, Anseth KS (2011). Cytocompatible click-based hydrogels with dynamically tunable properties through orthogonal photoconjugation and photocleavage reactions. Nat Chem.

[52] Kloxin AM, Kasko AM, Salinas CN, Anseth KS (2009). Photodegradable hydrogels for dynamic tuning of physical and chemical properties. Science.

[53] Ondeck MG, Engler AJ. Mechanical Characterization of a Dynamic and Tunable Methacrylated Hyaluronic Acid Hydrogel. J Biomech Eng. 2016;138. 10.1115/1.4032429 PMC4844086 26746491

[54] Young JL, Engler AJ (2011). Hydrogels with time-dependent material properties enhance cardiomyocyte differentiation in vitro. Biomaterials.

[55] Hribar KC, Choi YS, Ondeck M, Engler AJ, Chen S (2014). Digital Plasmonic Patterning for Localized Tuning of Hydrogel Stiffness. Adv Funct Mater.

[56] Purcell BP, Lobb D, Charati MB, Dorsey SM, Wade RJ, Zellars KN (2014). Injectable and bioresponsive hydrogels for on-demand matrix metalloproteinase inhibition. Nat Mater.

[57] Sydney Gladman A, Matsumoto EA, Nuzzo RG, Mahadevan L, Lewis JA (2016). Biomimetic 4D printing. Nat Mater.

[58] Park JS, Chu JS, Cheng C, Chen F, Chen D, Li S (2004). Differential effects of equiaxial and uniaxial strain on mesenchymal stem cells. Biotechnol Bioeng.

[59] Ward DF, Salasznyk RM, Klees RF, Backiel J, Agius P, Bennett K (2007). Mechanical strain enhances extracellular matrix-induced gene focusing and promotes osteogenic differentiation of human mesenchymal stem cells through an extracellular-related kinase-dependent pathway. Stem Cells Dev.

[60] Guvendiren M, Burdick JA (2013). Stem cell response to spatially and temporally displayed and reversible surface topography. Adv Healthc Mater.

[61] Charoenpanich A, Wall ME, Tucker CJ, Andrews DM, Lalush DS, Dirschl DR (2014). Cyclic tensile strain enhances osteogenesis and angiogenesis in mesenchymal stem cells from osteoporotic donors. Tissue Eng Part A.

[62] Wang X, Yan C, Ye K, He Y, Li Z, Ding J (2013). Effect of RGD nanospacing on differentiation of stem cells. Biomaterials.

[63] Tsai TL, Nelson BC, Anderson PA, Zdeblick TA, Li WJ (2014). Intervertebral disc and stem cells cocultured in biomimetic extracellular matrix stimulated by cyclic compression in perfusion bioreactor. Spine J.

[64] Steward AJ, Wagner DR, Kelly DJ (2013). The pericellular environment regulates cytoskeletal development and the differentiation of mesenchymal stem cells and determines their response to hydrostatic pressure. Eur Cell Mater.

[65] Prodanov L, van Loon JJ, te Riet J, Jansen JA, Walboomers XF (2013). Substrate nanotexture and hypergravity through centrifugation enhance initial osteoblastogenesis. Tissue Eng Part A.

[66] Uddin SM, Qin YX (2013). Enhancement of osteogenic differentiation and proliferation in human mesenchymal stem cells by a modified low intensity ultrasound stimulation under simulated microgravity. PLoS One.

[67] Liu L, Zong C, Li B, Shen D, Tang Z, Chen J (2014). The interaction between beta1 integrins and ERK1/2 in osteogenic differentiation of human mesenchymal stem cells under fluid shear stress modelled by a perfusion system. J Tissue Eng Regen Med.

[68] Gershlak JR, Resnikoff JI, Sullivan KE, Williams C, Wang RM, Black LD (2013). Mesenchymal stem cells ability to generate traction stress in response to substrate stiffness is modulated by the changing extracellular matrix composition of the heart during development. Biochem Biophys Res Commun.

[69] Ma J, He X, Jabbari E (2011). Osteogenic differentiation of marrow stromal cells on random and aligned electrospun poly(L-lactide) nanofibers. Ann Biomed Eng.

[70] Shanmugasundaram S, Chaudhry H, Arinzeh TL (2011). Microscale versus nanoscale scaffold architecture for mesenchymal stem cell chondrogenesis. Tissue Eng Part A.

[71] Rodrigues MT, Leonor IB, Groen N, Viegas CA, Dias IR, Caridade SG (2014). Bone marrow stromal cells on a three-dimensional bioactive fiber mesh undergo osteogenic differentiation in the absence of osteogenic media supplements: the effect of silanol groups. Acta Biomater.

[72] Lee J, Abdeen AA, Huang TH, Kilian KA (2014). Controlling cell geometry on substrates of variable stiffness can tune the degree of osteogenesis in human mesenchymal stem cells. J Mech Behav Biomed Mater.

[73] Zhong W, Zhang W, Wang S, Qin J (2013). Regulation of fibrochondrogenesis of mesenchymal stem cells in an integrated microfluidic platform embedded with biomimetic nanofibrous scaffolds. PLoS One.

[74] Volloch V, Olsen BR (2013). Why cellular stress suppresses adipogenesis in skeletal tissue, but is ineffective in adipose tissue: control of mesenchymal cell differentiation via integrin binding sites in extracellular matrices. Matrix Biol.

[75] Smith Callahan LA, Policastro GM, Bernard SL, Childers EP, Boettcher R, Becker ML (2013). Influence of discrete and continuous culture conditions on human mesenchymal stem cell lineage choice in RGD concentration gradient hydrogels. Biomacromolecules.

[76] Trappmann B, Gautrot JE, Connelly JT, Strange DG, Li Y, Oyen ML (2012). Extracellular-matrix tethering regulates stem-cell fate. Nat Mater.

[77] Murphy CM, Matsiko A, Haugh MG, Gleeson JP, O’Brien FJ (2012). Mesenchymal stem cell fate is regulated by the composition and mechanical properties of collagen-glycosaminoglycan scaffolds. J Mech Behav Biomed Mater.

[78] Flaim CJ, Chien S, Bhatia SN (2005). An extracellular matrix microarray for probing cellular differentiation. Nat Meth.

[79] He F, Liu X, Xiong K, Chen S, Zhou L, Cui W (2014). Extracellular matrix modulates the biological effects of melatonin in mesenchymal stem cells. J Endocrinol.

[80] Ranga A, Gobaa S, Okawa Y, Mosiewicz K, Negro A, Lutolf MP (2014). 3D niche microarrays for systems-level analyses of cell fate. Nat Commun.

[81] Dumas V, Ducharne B, Perrier A, Fournier C, Guignandon A, Thomas M (2010). Extracellular matrix produced by osteoblasts cultured under low-magnitude, high-frequency stimulation is favourable to osteogenic differentiation of mesenchymal stem cells. Calcif Tissue Int.

[82] Marinkovic M, Block TJ, Rakian R, Li Q, Wang E, Reilly MA et al. One size does not fit all: developing a cell-specific niche for in vitro study of cell behavior. Matrix Biol. 2016;52-54:426-41. 10.1016/j.matbio.2016.01.004 PMC5402896 26780725

[83] Pati F, Song TH, Rijal G, Jang J, Kim SW, Cho DW (2015). Ornamenting 3D printed scaffolds with cell-laid extracellular matrix for bone tissue regeneration. Biomaterials.

[84] Sadr N, Pippenger BE, Scherberich A, Wendt D, Mantero S, Martin I (2012). Enhancing the biological performance of synthetic polymeric materials by decoration with engineered, decellularized extracellular matrix. Biomaterials.

[85] Aamodt JM, Grainger DW (2016). Extracellular matrix-based biomaterial scaffolds and the host response. Biomaterials.

[86] Ahmed M, Ffrench-Constant C (2016). Extracellular Matrix Regulation of Stem Cell Behavior. Curr Stem Cell Rep.

[87] Radisic M, Christman KL (2013). Materials science and tissue engineering: repairing the heart. Mayo Clin Proc.

[88] Song JJ, Ott HC (2011). Organ engineering based on decellularized matrix scaffolds. Trends Mol Med.

[89] Choi YS, Vincent LG, Lee AR, Kretchmer KC, Chirasatitsin S, Dobke MK (2012). The alignment and fusion assembly of adipose-derived stem cells on mechanically patterned matrices. Biomaterials.

[90] De Lisio M, Jensen T, Sukiennik RA, Huntsman HD, Boppart MD (2014). Substrate and strain alter the muscle-derived mesenchymal stem cell secretome to promote myogenesis. Stem Cell Res Ther.

[91] Jeffords ME, Wu J, Shah M, Hong Y, Zhang G (2015). Tailoring material properties of cardiac matrix hydrogels to induce endothelial differentiation of human mesenchymal stem cells. ACS Appl Mater Interfaces.

[92] Young JL, Tuler J, Braden R, Schup-Magoffin P, Schaefer J, Kretchmer K (2013). In vivo response to dynamic hyaluronic acid hydrogels. Acta Biomater.

[93] Ifkovits JL, Tous E, Minakawa M, Morita M, Robb JD, Koomalsingh KJ (2010). Injectable hydrogel properties influence infarct expansion and extent of postinfarction left ventricular remodeling in an ovine model. Proc Natl Acad Sci U S A.

[94] Qin TW, Sun YL, Thoreson AR, Steinmann SP, Amadio PC, An KN (2015). Effect of mechanical stimulation on bone marrow stromal cell-seeded tendon slice constructs: a potential engineered tendon patch for rotator cuff repair. Biomaterials.

[95] Qiu Y, Lei J, Koob TJ, Temenoff JS. Cyclic tension promotes fibroblastic differentiation of human MSCs cultured on collagen-fibre scaffolds. J Tissue Eng Regen Med. 2014. 10.1002/term.1880 24515660

[96] Bosworth LA, Rathbone SR, Bradley RS, Cartmell SH (2014). Dynamic loading of electrospun yarns guides mesenchymal stem cells towards a tendon lineage. J Mech Behav Biomed Mater.

[97] Youngstrom DW, Rajpar I, Kaplan DL, Barrett JG (2015). A bioreactor system for in vitro tendon differentiation and tendon tissue engineering. J Orthop Res.

[98] Badylak SF, Brown BN, Gilbert TW, Daly KA, Huber A, Turner NJ (2011). Biologic scaffolds for constructive tissue remodeling. Biomaterials.

[99] Tong WY, Shen W, Yeung CW, Zhao Y, Cheng SH, Chu PK (2012). Functional replication of the tendon tissue microenvironment by a bioimprinted substrate and the support of tenocytic differentiation of mesenchymal stem cells. Biomaterials.

[100] Samavedi S, Olsen Horton C, Guelcher SA, Goldstein AS, Whittington AR (2011). Fabrication of a model continuously graded co-electrospun mesh for regeneration of the ligament-bone interface. Acta Biomater.

[101] Oortgiesen DA, Yu N, Bronckers AL, Yang F, Walboomers XF, Jansen JA (2012). A three-dimensional cell culture model to study the mechano-biological behavior in periodontal ligament regeneration. Tissue Eng Part C Methods.

[102] Angele P, Yoo JU, Smith C, Mansour J, Jepsen KJ, Nerlich M (2003). Cyclic hydrostatic pressure enhances the chondrogenic phenotype of human mesenchymal progenitor cells differentiated in vitro. J Orthop Res.

[103] Huang CY, Hagar KL, Frost LE, Sun Y, Cheung HS (2004). Effects of cyclic compressive loading on chondrogenesis of rabbit bone-marrow derived mesenchymal stem cells. Stem Cells.

[104] Luo ZJ, Seedhom BB (2007). Light and low-frequency pulsatile hydrostatic pressure enhances extracellular matrix formation by bone marrow mesenchymal cells: an in-vitro study with special reference to cartilage repair. Proc Inst Mech Eng H.

[105] Schumann D, Kujat R, Nerlich M, Angele P (2006). Mechanobiological conditioning of stem cells for cartilage tissue engineering. Biomed Mater Eng.

[106] Terraciano V, Hwang N, Moroni L, Park HB, Zhang Z, Mizrahi J (2007). Differential response of adult and embryonic mesenchymal progenitor cells to mechanical compression in hydrogels. Stem Cells.

[107] Schumann D, Kujat R, Zellner J, Angele MK, Nerlich M, Mayr E (2006). Treatment of human mesenchymal stem cells with pulsed low intensity ultrasound enhances the chondrogenic phenotype in vitro. Biorheology.

[108] Elder SH, Shim JW, Borazjani A, Robertson HM, Smith KE, Warnock JN (2008). Influence of hydrostatic and distortional stress on chondroinduction. Biorheology.

[109] Schatti O, Grad S, Goldhahn J, Salzmann G, Li Z, Alini M (2011). A combination of shear and dynamic compression leads to mechanically induced chondrogenesis of human mesenchymal stem cells. Eur Cell Mater.

[110] Vaughan TJ, Voisin M, Niebur GL, McNamara LM. Multiscale modeling of trabecular bone marrow: understanding the micromechanical environment of mesenchymal stem cells during osteoporosis. J Biomech Eng. 2015;137. 10.1115/1.4028986 25363305

[111] Di Luca A, Ostrowska B, Lorenzo-Moldero I, Lepedda A, Swieszkowski W, Van Blitterswijk C (2016). Gradients in pore size enhance the osteogenic differentiation of human mesenchymal stromal cells in three-dimensional scaffolds. Sci Rep.

[112] Becquart P, Cruel M, Hoc T, Sudre L, Pernelle K, Bizios R (2016). Human mesenchymal stem cell responses to hydrostatic pressure and shear stress. Eur Cell Mater.

[113] Sharp LA, Lee YW, Goldstein AS (2009). Effect of low-frequency pulsatile flow on expression of osteoblastic genes by bone marrow stromal cells. Ann Biomed Eng.

[114] Chen X, He F, Zhong DY, Luo ZP (2015). Acoustic-frequency vibratory stimulation regulates the balance between osteogenesis and adipogenesis of human bone marrow-derived mesenchymal stem cells. Biomed Res Int.

[115] Emani S, Mayer JE, Emani SM (2011). Gene regulation of extracellular matrix remodeling in human bone marrow stem cell-seeded tissue-engineered grafts. Tissue Eng Part A.

[116] Joiner DM, Tayim RJ, Kadado A, Goldstein SA (2012). Bone marrow stromal cells from aged male rats have delayed mineralization and reduced response to mechanical stimulation through nitric oxide and ERK1/2 signaling during osteogenic differentiation. Biogerontology.

[117] Iafiscol M, Quirici N, Foltran I, Rimondini L (2013). Electrospun collagen mimicking the reconstituted extracellular matrix improves osteoblastic differentiation onto titanium surfaces. J Nanosci Nanotechnol.

